# The mitochondrial genome of *Binodoxys acalephae* (Hymenoptera: Braconidae) with unique gene rearrangement and phylogenetic implications

**DOI:** 10.1007/s11033-022-08232-0

**Published:** 2023-01-13

**Authors:** Shiwen Xu, Weiwei Li, Qiannan Liu, Yunming Wang, Xiaoling Li, Xiaoqian Duan, Jia He, Fan Song

**Affiliations:** 1 Department of Entomology and MOA Key Lab of Pest Monitoring and Green Management, College of Plant Protection, China Agricultural University, 100193 Beijing, China; 2 Kunming Institute of Zoology, Chinese Academy of Sciences, 650223 Kunming, China; 3 Yunnan Agricultural University, 650201 Kunming, China; 4 Yuxi Branch, Yunnan Tobacco Company, 653100 Yuxi, China; 5 Institute of Plant Protection, Academy of Ningxia Agriculture and Forestry Science, 750002 Yinchuan, China; 6 Ningxia Key Laboratory of Plant Disease and Pest Control, 750002 Yinchuan, China

**Keywords:** *Binodoxys acalephae*, Mitochondrial genome, Gene rearrangement, Phylogeny

## Abstract

**Background:**

Species in the subfamily Aphidiinae from the Braconidae of Hymenoptera are endoparasitic wasps that exclusively utilize aphids as hosts. Some Aphidiinae species are widely used as biological agents. However, there were only one species with determined complete mitochondrial genome from this subfamily.

**Methods and results:**

In this study, we sequenced and annotated the mitochondrial genome (mitogenome) of *Binodoxys acalephae*, which was 15,116 bp in size and contained 37 genes. The start codon of 13 protein-coding genes was ATN, and the complete stop codon TAA and TAG was widely assigned to 11 protein-coding genes. The *lrRNA* contains 43 stem-loop structures, and *srRNA* contains 25 stem-loop structures. Translocation and inversion of tRNA genes was found to be dominant in *B. acalephae*. In contrast to *Aphidius gifuensis* from the same subfamily Aphidiinae, inverted *tRNA*^*Leu1*^ was translocated to the gene cluster between *tRNA*^*Leu2*^ and *COX2*, and the control region between *tRNA*^*Ile*^ and *tRNA*^*Met*^ was deleted in the mitogenome of *B. acalephae*. Within Braconidae, gene clusters *tRNA*^*Trp*^-*tRNA*^*Cys*^*-tRNA*^*Tyr*^ and CR-*tRNA*^*Ile*^*-tRNA*^*Gln*^*-tRNA*^*Met*^ were hotspots for gene rearrangement. Phylogenetic analysis showed that both Bayesian and maximum-likelihood methods recovered the monophyly of Aphidiinae and suggested that Aphidiinae formed sister clades with the remaining subfamilies. The phylogenetic analyses of nine subfamilies supported the monophyly of Cyclostomes and Noncyclostomes in Braconidae.

**Conclusion:**

The arrangement of mitochondrial genes and the phylogenetic relationships among nine Braconidae subfamilies were constructed better to understand the diversity and evolution of Aphidiinae mitogenomes.

**Supplementary information:**

The online version contains supplementary material available at 10.1007/s11033-022-08232-0.

## Introduction

Insect mitochondrial genome (mitogenome) is a double-stranded circular DNA molecule, which measures approximately 14–20 kb in size and contains 13 protein-coding genes (PCGs), 22 transfer RNA (tRNA) genes, two ribosomal RNA (rRNA) genes, and a control region (CR) [1, 2]. Due to its small size, simple structure, and rapid evolutionary rate, mitogenome has been extensively studied and widely used for species identification, molecular phylogeny, phylogeography, and molecular evolutionary research [3–5]. Although the arrangement of the mitochondrial genes is generally conserved across most inset orders [1, 6], gene rearrangement is also quite common in some highly derived groups [4, 7–10]. Hymenoptera (bees, wasps, hornets, sawflies, and ants) is one of the most important groups for studying mitogenome rearrangements in insects, with at least one translocated tRNA in each hymenopteran mitogenome [11, 12]. The mitogenomes of Hymenoptera are characterized by high A + T content [13], high substitution rates [14] and strong base composition bias [15], which may accelerate the rate of gene rearrangement. Mitochondrial gene rearrangement patterns were commonly used to revise the phylogeny in the species-rich family Braconidae, which was traditionally divided into Cyclostomes and Noncyclostomes in previous studies [16–20].

The species from the subfamily Aphidiinae (Hymenoptera: Braconidae) are endoparasitic wasps that exclusively utilize aphids (Hemiptera) as hosts. The adults of Aphidiinae lay eggs inside the aphids, and the larva gradually consumes the soft tissue of the hosts and spin a cocoon within them, forming a characteristic “mummy” [21]. Therefore, some species of wasps are widely used as biological control agents of aphids [22, 23]. This subfamily currently includes 56 genera and more than 500 species worldwide [24]. For a long time, Aphidiinae was considered to be a separate family “Aphidiidae”, located between the Cyclostomes and Noncyclostomes [20, 25, 26]. However, recent studies supported that Aphidiinae was a subfamily placed within the complex Cyclostomes [17, 27, 28]. Up to now, the complete mitogenome of only one species (*Aphidius gifuensis*) has been sequenced and annotated in the Aphidiinae [18]. In this study, the mitogenome of *Binodoxys acalephae* was sequenced and described, and the phylogenetic relationships among major Braconidae subfamilies were constructed to better understand the diversity and evolution of Aphidiinae mitogenomes.

## Materials and methods

### Sample collection and DNA extraction

Adult specimen of *B. acalephae* was collected from Kunming, Yunnan Province. The specimen was preserved in anhydrous ethanol and stored in a refrigerator at -20℃ before DNA extraction. After being identified based on morphological characters, the genomic DNA was extracted using DNeasy Blood and Tissue kit (Qiagen, Germany) on the basis of the manufacturer’s protocol, and the voucher specimen was stored at Entomological Museum of China Agricultural University.

### Mitogenome sequencing and assembly

The Illumina TruSeq library was prepared with an average insert size of 350 bp and sequenced with the paired-end reads length of 150 bp on Illumina NovaSeq 6000 platform (Berry Genomic, Beijing, China). A total of 6 Gb raw data was obtained, and use Prinseq version 0.20.4 [29] to remove short and low-quality reads with the poly-Ns > 15 bp, or > 75 bp bases with quality score < 3. The remaining reads were *de novo* assembled using IDBA-UD [30], with minimum and maximum k values of 41 and 141 bp, respectively. The *COX1* fragment (~ 610 bp) was amplified by PCR reaction (forward primer, 5’-3’: GGTCAACAAATCATAAAGATATTGG; reverse primer, 5’-3’: TAAACTTCAGGGTGACCAAAAAATCA). PCR cycling condition was: 95℃ for 1 min, 40 cycles of 95℃ for 20s, 50℃ for 50s, and 68℃ for 1.5 min, followed by 72℃ for 5 min. The PCR products were sequenced by Sanger sequencing at Tsingke Biotechnology (Beijing, China). To identify the corresponding mitogenome assemblies, the assembled contigs were searched with *COX1* sequence using BLAST with at least 98% similarity. To investigate the assembled accuracy and sequencing depth, clean reads were mapped using Geneious version 10.1.3 [31]. Finally, we obtained a complete circular mitogenome with an average coverage depth of 676×.

### Mitogenome annotation and nucleotide composition analysis

The gene sequences were preliminarily annotated by MitoZ [32] and accurately corrected in Geneious. The locations and secondary structures of tRNA genes were mainly determined by tRNAscan-SE search server [33]and ARWEN version 1.2 [34]. PCGs and rRNA genes were identified by alignment with homologous genes of other Braconidae species, and the secondary structure of rRNAs was predicted by RNAfold WebServer Version 2.4.18 [35] online platform. The gene rearrangement of *B. acalephae* was compared with the putative ancestral type of *Drosophila melanogaster* and 15 other published Braconidae species (Table 1). The nucleotide composition of mitogenome and synonymous codon usage (RSCU) of PCGs were analyzed by MEGA version 7.0 [36]. Nucleotide compositional differences were calculated by the formulae: AT skew = (A – T) / (A + T) and GC skew = (G - C) / (G + C) [37].

**Table 1:** Mitogenomes used in this study

Group	Family	Subfamily	Species	GenBank accession number
Outgroup	Ichnummonidae	Campopleginae	*Diadegma semiclausum*	EU871947
		Ophioninae	*Enicospilus* sp.	FJ478177
Ingroup	Braconidae	Aphidiinae	*Aphidius gifuensis*	MT264907
		Aphidiinae	Binodoxys acalephae*	OP612694
		Alysiinae	*Asobara japonica*	MN882556
		Braconinae	*Euurobracon yokohamae*	OL825724
		Braconinae	*Habrobracon hebetor*	MN842279
		Cardiochilinae	*Cardiochiles fuscipennis*	KF385870
		Cheloninae	*Chelonus formosanus*	OK299152
		Doryctinae	*Spathius agrili*	NC_014278
		Euphorinae	*Meteorus pulchricornis*	MK907921
		Euphorinae	*Zele chlorophthalmus*	NC_039181
		Microgastrinae	*Cotesia vestalis*	NC_014272
		Opiinae	*Fopius arisanus*	MZ128286
		Opiinae	*Psyttalia concolor*	MW279212
		Opiinae	*Psyttalia humilis*	MW279213
		Opiinae	*Psyttalia incisi*	OK413636
		Opiinae	*Psyttalia lounsburyi*	MW279214

* Newly sequenced mitogenome

### Phylogenetic analysis

The phylogenetic analysis was based on newly sequenced mitogenome of *B. acalephae* together with 15 mitogenomes published in GenBank from nine main subfamilies of Braconidae. *Diadegma semiclausum* and *Enicospilus* sp. from Ichneumonidae were selected as outgroups (Table 1).

The 13 PCGs of each species were aligned separately using the L-INS-I strategy of the MAFFT algorithm [38] implemented in TranslatorX [39]. Two rRNA genes were aligned individually using the G-INS-I strategy of MAFFT version 7.0 online server [40]. All alignments were checked manually in MEGA [36]. Gene fragments were imported into Geneious for concatenation into two datasets: (1) the PCGRNA matrix with 10,782 nucleotides including 13 PCGs and two rRNA genes (*srRNA* and *lrRNA*); (2) the PCG12RNA matrix with 7,430 nucleotides including the first and second codon positions of the 13 PCGs and two rRNA genes.

These two datasets were analyzed under the strategy of Bayesian inference (BI) and Maximum-likelihood (ML). The BI phylogenetic trees were recovered using PhyloBayes MPI version 1.5a [41] under the site-heterogeneous mixture CAT + GTR model [42, 43]. The Markov Chain Monte Carlo chains were run independently after removing constant sites from the alignment and were stopped after the two runs had satisfactorily converged (maxdiff < 0.1). A consensus tree was yielded from the remaining trees after discarding initial 25% trees of each run as burn-in. The ML phylogenetic trees were recovered using IQ-TREE web server [44], with automatic model prediction and1000 SH-aLRT replicates.

## Results

### Genome organization and base composition

**Fig. 1:**
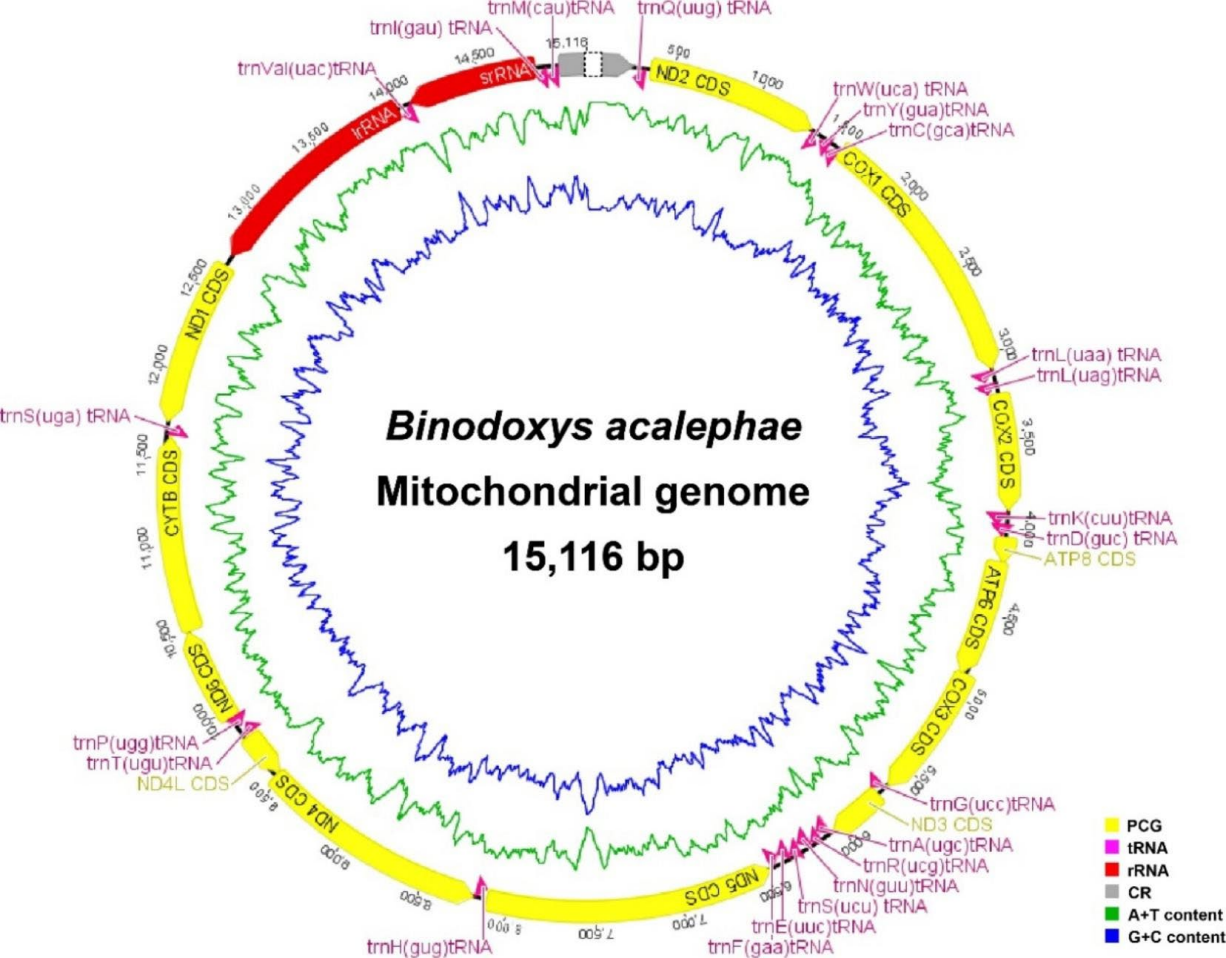
The mitochondrial genome of *Binodoxys acalephae*. Orientation of gene transcription is indicated by the arrows. Protein-coding genes (PCGs) are shown as yellow arrows, transfer RNA (tRNA) genes as pink arrows, ribosomal RNA (rRNA) genes as red arrows. The partial control region (in grey) contains incomplete repeated region. The green line in the circle shows the A + T content, and the blue shows the G + C content

We obtained a nearly complete mitogenome of *B. acalephae* (GenBank accession number OP612694), which was 15,116 bp in size and included all the 37 protein-coding genes (13 PCGs, 22 tRNAs and two rRNAs) and a partial control region with the exact number of the tandem repeated units undetermined (Fig. 1). Four PCGs (*ND5*, *ND4*, *ND4L* and *ND1*), nine tRNA genes (*tRNA*^*Gln*^, *tRNA*^*Tyr*^, *tRNA*^*Cys*^, *tRNA*^*Phe*^, *tRNA*^*His*^, *tRNA*^*Pro*^, *tRNA*^*Val*^, *tRNA*^*Ile*^ and *tRNA*^*Met*^) and two rRNA genes (*srRNA* and *lrRNA*) were encoded on the minority strand (N-strand) while the other 22 genes were encoded on the majority strand (J-strand). In the mitogenome of *B. acalephae*, 14 gene overlap regions with a total of 48 bp were observed, ranging from 1 to 11 bp in size, and the longest gene overlap region was between *ATP6* and *COX3*. Apart from the control region, we identified 13 non-coding regions (NCRs) comprising a total of 127 bp, with the longest NCR (56 bp) located between *ND1* and *lrRNA* (Table S1).

The A + T content of the whole mitogenome of *B. acalephae* was 82.7% and the mitogenome of *B. acalephae* shows a negative AT skew (-0.115) and a positive GC skew (0.179), indicating that the nucleotide composition was significantly biased toward T (Table S2).

### PCGs and codon usage

All 13 PCGs were arranged in putative ancestral gene order in the mitogenome of *B. acalephae*. The total length of these PCGs was 11,138 bp, and the A + T content of PCGs (81.0%) was slightly lower than that of the whole mitogenome (82.7%). We analyzed the nucleotide composition of each codon position in PCGs and found that A or T are overwhelmingly overrepresented at the third codon position on each strand (A + T %= 86.2%). The negative AT skew was consistent in all three codons, while the positive GC skew only existed in the first (0.212) and third (0.029) codons (Table S2).

The bias toward A and T of *B. acalephae* mitogenome was also well documented in the table of codon usage (Table S3). We calculated the relative synonymous codon usage (RSCU) (Fig S1), and found the six most prevalent codons, UUA, GCU, CGU, ACU, GUU and UCU were mainly composed by T and/or A. At the third codon position, A or T were overrepresented in PCGs.

All 13 PCGs were initiated with ATN as the start codon (five with ATT, six with ATG, and two with ATA) (Table S1). The complete stop codon TAA and TAG was most widely assigned to ten PCGs and *CYTB*, respectively, while *COX2* and *ND3* used a single T residue as an incomplete stop codon.

### tRNA genes and rRNA genes

Twenty-two tRNAs were detected in the mitogenome of *B. acalephae*, ranging from 62 bp (*tRNA*^*Cys*^ and *tRNA*^*Thr*^) to 71 bp (*tRNA*^*Lys*^) in size. Different from the ancestral tRNA arrangement, there were 13 tRNAs encoded by J-strand and nine encoded by N-strand. Among all tRNAs, 21 tRNAs of *B. acalephae* could be folded into the typical cloverleaf secondary structures except for *tRNA*^*Ser(AGN)*^ (Fig S2). The dihydrouridine (DHU) arm of *tRNA*^*Ser(AGN)*^ was a simple loop. Unlike the variable DHU-stem (3–4 bp) and TΨC‐stem (3–6 bp), the anticodon stem (5 bp) and amino-acid acceptor stem (7 bp) are relatively conservative in size, except for *tRNA*^*Gln*^ with amino-acid acceptor (8 bp) stem and *tRNA*^*Ser(AGN)*^ with anticodon stem (6 bp). A total of 23 mismatched base pairs were detected in 15 tRNAs with 15 G‐U pairs and 8 U‐U pair.

**Fig. 2:**
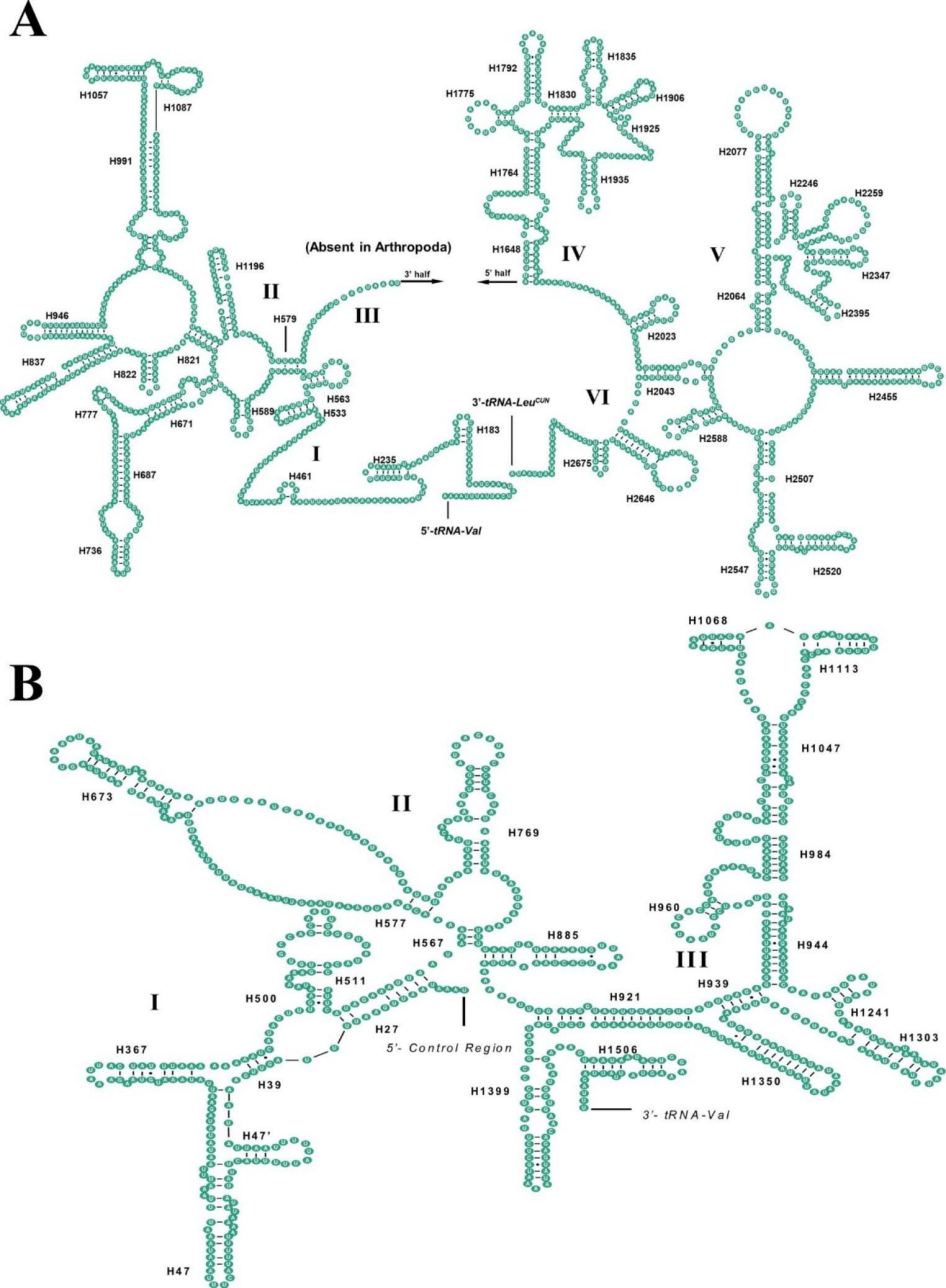
Predicted secondary structures of the *lrRNA* (A) and *srRNA* (B) of *Binodoxys acalephae*. Short lines indicate Watson–Crick base-pairing and dots indicate noncanonical G-U pairs

*lrRNA* and *srRNA* were 1,283 bp and 745 bp in size, respectively, with an average A + T content of 86.2% (Table S2). We predicted the secondary structures for rRNAs using the RNAfold WebServer online platform and homologous species alignment. We identified that *lrRNA* has six domains (Fig. 2 A), among which the IV and V domains are relatively conserved, and the III domain only has a short sequence compared with other species. There were three domains in *srRNA* (Fig. 2B), and the III domain was more conserved than the I and II domains. Overall, *lrRNA* contains 43 stem-loop structures, and *srRNA* contains 25 stem-loop structures.

### Gene rearrangement

**Fig. 3:**
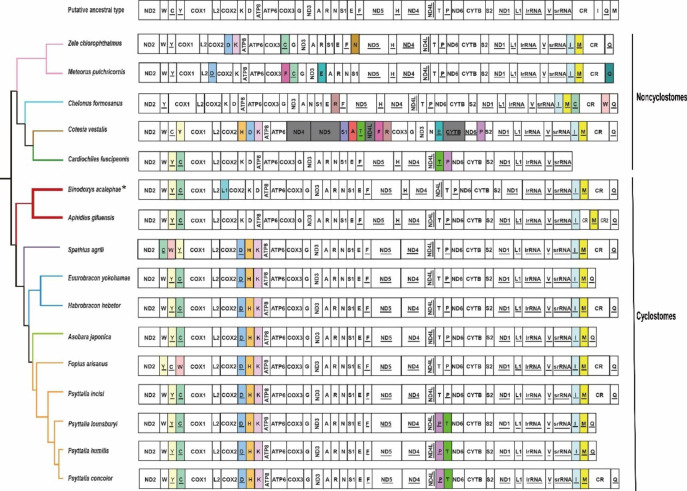
Mitochondrial genome organization in Braconidae referenced with the ancestral insect mitochondrial genomes. The topology is derived from the following phylogenetic analyses. The control region is abbreviated as CR and tRNA genes are denoted by a one-letter symbol according to the IPUC-IUB single-letter amino acid codes. Genes with underlines indicate that the gene is encoded on the minority strand. Rearranged protein-coding genes are marked in grey, and each of the rearranged tRNAs is marked with a different color

Gene rearrangements could be classified into translocations, local inversions (inverted in the local position), gene shuffling (local translocation) and remote inversions (translocated and inverted) [12]. Compared with the putative ancestral gene arrangement of *D. melanogaster*, there were at least seven tRNA rearrangement events in *B. acalephae*. These gene rearrangements were mainly from the tRNA clusters of *tRNA*^*Trp(W)*^*-tRNA*^*Cys(C)*^*-tRNA*^*Tyr(Y)*^ and CR-*tRNA*^*Ile(I)*^*-tRNA*^*Gln(Q)*^*-tRNA*^*Met(M)*^ (Fig. 3). The gene shuffling of *tRNA*^*Cys(C)*^ and *tRNA*^*Tyr(Y)*^, and remote inversions of *tRNA*^*Ile(I)*^, *tRNA*^*Met(M)*^ and *tRNA*^*Leu1(L1)*^ were found in the mitogenome of *B. acalephae*.

### Phylogenetic analyses

**Fig. 4:**
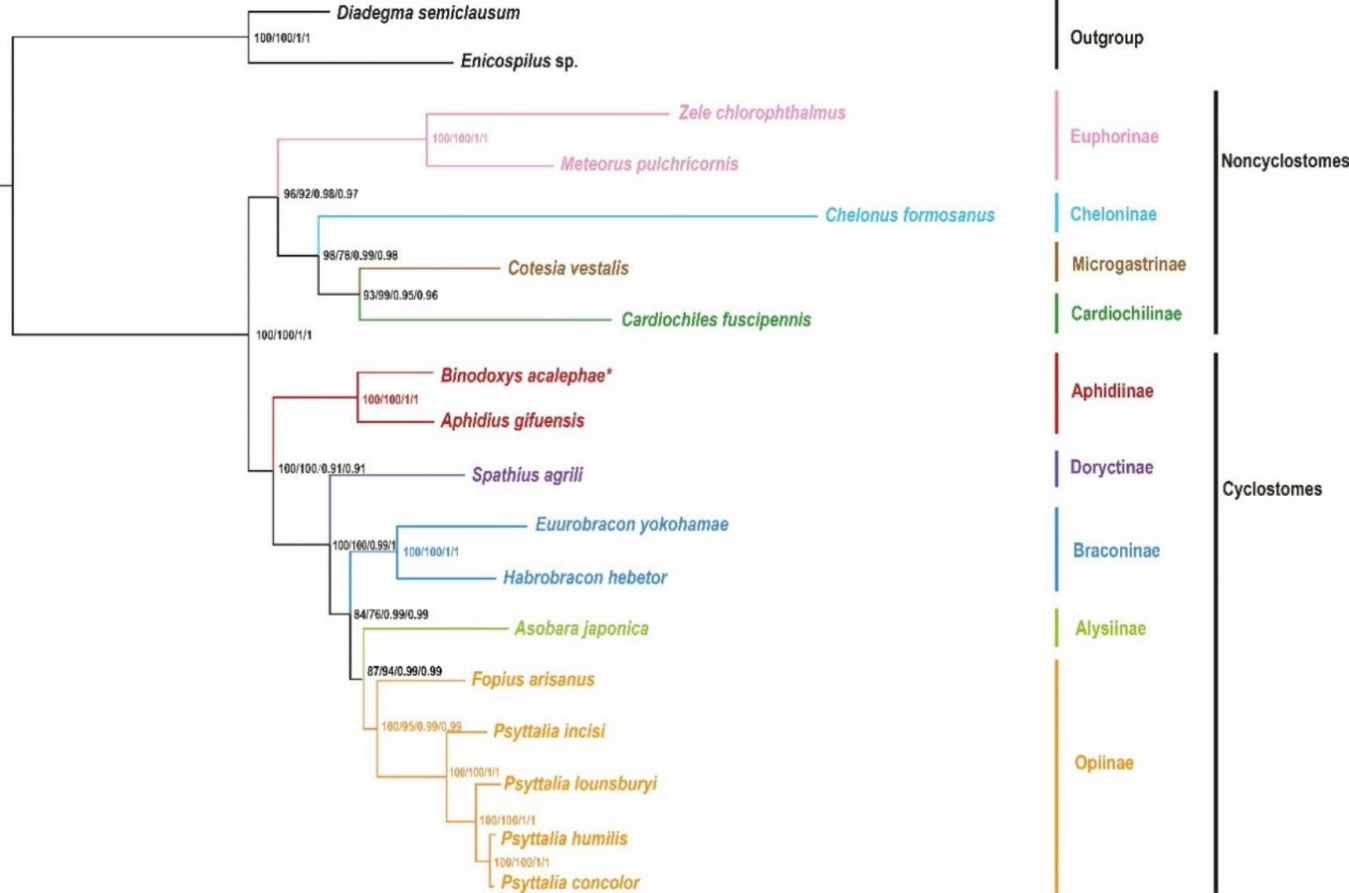
Phylogenetic trees inferred from Bayesian inference and maximum-likelihood analyses of the PCGRNA and PCG12RNA datasets. Values at corresponding nodes are ML bootstrap values (BSV) for PCGRNA, PCG12RNA and Bayesian posterior probabilities (BPP) for PCGRNA, PCG12RNA from left to right, respectively

Phylogenetic studies were conducted on 16 mitogenomes of Braconidae by using two species of Ichnummonidae as outgroups. PhyloBayes and ML analyses based on the two datasets (PCGRNA and PCG12RNA) yield identical topology with high node support values among subfamilies in Braconidae. Our analyses supported the monophyly of the subfamily Aphidiinae with high Bayesian posterior probabilities (BPP = 1) and ML bootstrap values (BSV = 100). The subfamily Aphidiinae formed sister clades with the remaining subfamilies (BPP = 0.91 and BSV = 100) (Fig. 4).

## Discussion

The mitogenome of *B. acalephae* was highly conserved in terms of gene content, gene size, base composition, PCG codon usage, and RNA secondary structures. In terms of base composition, the high A + T content of *B. acalephae* was corresponding with that of hymenopteran mitogenomes [14, 45, 46]. In terms of PCG codon usage, the most prevalent codons were mainly composed by T and/or A, which is consistent with other hymenopteran mitogenomes [4, 15, 16, 47]. And a single T residue as an incomplete stop codon has been noticed in several other hymenopteran mitogenomes [48–50]. In terms of RNA secondary structures, the simple loop of DHU arm of *tRNA*^*Ser(AGN)*^ was found in many other insects [2]. Compared with *A. gifuensis* from the same subfamily Aphidiinae, the mitochondrial gene of *B. acalephae* undergone two rearrangement events. The inverted *tRNA*^*Leu1*^ was translocated to the gene cluster between *tRNA*^*Leu2*^ and *COX2*, and the CR between *tRNA*^*Ile*^ and *tRNA*^*Met*^ was then deleted in the mitogenome of *B. acalephae*. Based on the topology derived from our phylogenetic analysis, we conclude five tRNA rearrangement events (R1 to R5) in Cyclostomes. The putative ancestral type rearranged to the gene arrangement of *A. gifuensis* by the tandem duplication-random loss [51] of gene block CR-*tRNA*^*Ile*^*-tRNA*^*Gln*^*-tRNA*^*Met*^ (R1). The CR between *tRNA*^*Ile*^ and *tRNA*^*Met*^ was lost in the remaining Cyclostomes species during evolution (R2). Then, *tRNA*^*Asp*^ was inverted and translocated upstream of *tRNA*^*Lys*^ (R3) and *tRNA*^*His*^ was remote inverted to form a gene cluster *tRNA*^*Asp*^-*tRNA*^*His*^-*tRNA*^*Lys*^ (R4). Last, *tRNA*^*Thr*^ and *tRNA*^*Pro*^ translocated in some species of the genus *Psyttalia* (R5). Furthermore, the ancestral gene arrangement of PCGs was present in all mitogenomes of Braconidae except for the *Cotesia vestalis* [16], suggesting heterogeneity in gene rearrangement rates among different Braconidae lineages. Gene clusters *tRNA*^*Trp*^*-tRNA*^*Cys*^*-tRNA*^*Tyr*^ and CR-*tRNA*^*Ile*^*-tRNA*^*Gln*^*-tRNA*^*Met*^ were found to be the gene rearrangement hotspots, in which remote translocation of *tRNA*^*Ile*^ and *tRNA*^*Met*^ may be synapomorphic rearrangements in Braconidae. The local inversion was reported to be the major type of gene rearrangement in Hymenoptera [11], while remote inversion of tRNA genes was found to be dominant in *B. acalephae*. This phenomenon was also found in other species of Braconidae and was thought to be the result of two independent recombination events [16].

In our analyses of nine subfamilies, the phylogeny supported the monophyly of Cyclostomes and Noncyclostomes. Phylogenetic analysis recovered the monophyly of Aphidiinae and suggested that Aphidiinae was the earliest branching lineage of Cyclostomes. The results were consistent with previous studies based on both mitochondrial and nuclear genes [20, 21]. Within the Noncyclostomes lineage, the sister relationship between Euphorinae and the remaining subfamilies of Noncyclostomes was recovered (Fig. 4).

## Electronic supplementary material

Below is the link to the electronic supplementary material.

Supplementary Material 1

## Data Availability

The data that support the findings of this study will be available in GenBank at https://www.ncbi.nlm.nih.gov/, with accession number OP612694.
