# Supplementary material for: The mitochondrial genome of Binodoxys acalephae (Hymenoptera: Braconidae) with unique gene rearrangement and phylogenetic implications
Source: Mol Biol Rep. 2023 Jan 13;50(3):2641–9. doi: 10.1007/s11033-022-08232-0 (PMC10011326; doi:10.1007/s11033-022-08232-0)
Supplement: Supplementary file 1 — Supplementary Material 1 [file 11033_2022_8232_MOESM1_ESM.docx]

Table S1. Organization of the mitochondrial genome of *Binodoxys acalephae*.

| Name | Direction | Location | Size | Anticodon | Codon | | Intergenic nucleotides |
| --- | --- | --- | --- | --- | --- | --- | --- |
|  |  |  |  |  | Start | Stop |  |
| *tRNA^Gln^* | R | 339-270 | 70 | 321-319TTG |  |  |  |
| *ND2* | F | 364-1353 | 990 |  | ATA | TAA | 25 |
| *tRNA^Trp^* | F | 1353-1418 | 66 | 1384-1386TCA |  |  | -1 |
| *tRNA^Tyr^* | R | 1484-1418 | 67 | 1454-1452GTA |  |  | -2 |
| *tRNA^Cys^* | R | 1545-1484 | 62 | 1516-1514GCA |  |  | 1 |
| *COX1* | F | 1550-3091 | 1542 |  | ATG | TAA | 3 |
| *tRNA^Leu^*^(^*^UUR^*^)^ | F | 3096-3161 | 66 | 3128-3130TAA |  |  | 4 |
| *tRNA^Leu^*^(^*^CUN^*^)^ | F | 3167-3231 | 63 | 3198-3200TAG |  |  | 6 |
| *COX2* | F | 3231-3906 | 676 |  | ATT | T | 0 |
| *tRNA^Lys^* | F | 3913-3983 | 71 | 3942-3944TTT |  |  | 6 |
| *tRNA^Asp^* | F | 3983-4048 | 66 | 4014-4016GTC |  |  | -1 |
| *ATP8* | F | 4049-4207 | 159 |  | ATT | TAA | 0 |
| *ATP6* | F | 4198-4878 | 681 |  | ATG | TAA | -10 |
| *COX3* | F | 4868-5650 | 783 |  | ATG | TAA | -11 |
| *tRNA^Gly^* | F | 5653-5717 | 63 | 5684-5686TCC |  |  | 3 |
| *ND3* | F | 5717-6068 | 352 |  | ATT | T | 0 |
| *tRNA^Ala^* | F | 6068-6132 | 63 | 6099-6101TGC |  |  | 0 |
| *tRNA^Arg^* | F | 6130-6197 | 66 | 6159-6161TCG |  |  | -1 |
| *tRNA^Asn^* | F | 6199-6265 | 65 | 6230-6232GTT |  |  | 3 |
| *tRNA^Ser^*^(^*^AGN^*^)^ | F | 6265-6330 | 66 | 6290-6292TCT |  |  | 0 |
| *tRNA^Glu^* | F | 6330-6396 | 67 | 6363-6365TTC |  |  | -1 |
| *tRNA^Phe^* | R | 6461-6397 | 65 | 6429-6427GAA |  |  | -1 |
| *ND5* | R | 8130-6460 | 1671 |  | ATT | TAA | -1 |
| *tRNA^His^* | R | 8195-8131 | 63 | 8163-8161GTG |  |  | 0 |
| *ND4* | R | 9540-8194 | 1347 |  | ATG | TAA | 0 |
| *ND4L* | R | 9818-9534 | 285 |  | ATT | TAA | -7 |
| *tRNA^Thr^* | F | 9821-9883 | 62 | 9851-9853TGT |  |  | 2 |
| *tRNA^Pro^* | R | 9948-9883 | 66 | 9917-9915TGG |  |  | -1 |
| *ND6* | F | 9951-10505 | 555 |  | ATG | TAA | 3 |
| *CYTB* | F | 10498-11637 | 1140 |  | ATG | TAG | -8 |
| *tRNA^Ser^*^(^*^UCN^*^)^ | F | 11636-11701 | 66 | 11665-11667TGA |  |  | -2 |
| *ND1* | R | 12663-11707 | 957 |  | ATA | TAA | 5 |
| *lrRNA* | R | 14002-12720 | 1283 |  |  |  | 56 |
| *tRNA^Val^* | R | 14078-14013 | 64 | 14046-14044TAC |  |  | 10 |
| *srRNA* | R | 14821-14075 | 745 |  |  |  | 0 |
| *tRNA^Ile^* | R | 14885-14822 | 64 | 14855-14853GAT |  |  | 0 |
| *tRNA^Met^* | R | 14950-14886 | 65 | 14917-14915CAT |  |  |  |

Table S2. Nucleotide composition and skewness of mitochondrial genome of *Binodoxys acalephae*.

| Feature | T(U) | C | A | G | A+T% | AT Skew | GC Skew |
| --- | --- | --- | --- | --- | --- | --- | --- |
| Whole genome | 46.1 | 7.1 | 36.6 | 10.2 | 82.7 | -0.115 | 0.179 |
| Protein-coding genes | 46.7 | 8.8 | 34.3 | 10.2 | 81.0 | -0.153 | 0.074 |
| First codon position | 42.3 | 7.8 | 37.9 | 12.0 | 80.2 | -0.055 | 0.212 |
| Second codon position | 50.6 | 11.7 | 26.0 | 11.6 | 76.6 | -0.321 | -0.004 |
| Third codon position | 47.3 | 6.7 | 38.9 | 7.1 | 86.2 | -0.097 | 0.029 |
| Protein-coding genes J-strand | 49.3 | 7.6 | 30.9 | 12.2 | 80.2 | -0.229 | 0.232 |
| First codon position | 46.1 | 7.2 | 33.5 | 13.2 | 79.6 | -0.158 | 0.294 |
| Second codon position | 49.7 | 9.3 | 28.4 | 12.6 | 78.1 | -0.273 | 0.151 |
| Third codon position | 52.0 | 6.3 | 30.8 | 11.0 | 82.8 | -0.256 | 0.272 |
| Protein-coding genes N-strand | 42.7 | 10.6 | 39.7 | 7.0 | 82.4 | -0.036 | -0.205 |
| First codon position | 36.1 | 8.9 | 44.9 | 10.1 | 81.0 | 0.109 | 0.063 |
| Second codon position | 52.1 | 15.6 | 22.2 | 10.1 | 74.3 | -0.402 | -0.214 |
| Third codon position | 39.8 | 7.3 | 52.0 | 0.8 | 91.8 | 0.133 | -0.802 |
| tRNA genes | 43.0 | 5.1 | 43.7 | 8.3 | 86.7 | 0.008 | 0.239 |
| tRNA genes J-strand | 43.9 | 4.8 | 42.9 | 8.4 | 86.8 | -0.012 | 0.273 |
| tRNA genes N-strand | 41.6 | 5.5 | 44.7 | 8.2 | 86.3 | 0.036 | 0.197 |
| rRNA genes | 37.9 | 6.5 | 48.3 | 7.3 | 86.2 | 0.121 | 0.058 |

Table S3. Codon number and RSCU of mitochondrial genome of *Binodoxys acalephae*. The underlined codons stand for the cognate codon of tRNA for each amino acid, and the values in bold type stand for the most commonly used codon for the amino acid.

|  | Codon | Count | RSCU |  | Codon | Count | RSCU |
| --- | --- | --- | --- | --- | --- | --- | --- |
| Phe | UUU(F) | 405 | **1.75** | Tyr | UAU(Y) | 286 | **1.68** |
|  | UUC(F) | 57 | 0.25 |  | UAC(Y) | 54 | 0.32 |
| Leu(UUR) | UUA(L) | 322 | **3.69** |  | UAA(*) | 281 | 1.61 |
|  | UUG(L) | 70 | 0.80 |  | UAG(*) | 69 | 0.39 |
| Leu(CUN) | CUU(L) | 51 | **0.58** | His | CAU(H) | 71 | **1.75** |
|  | CUC(L) | 21 | 0.24 |  | CAC(H) | 10 | 0.25 |
|  | CUA(L) | 44 | 0.50 | Gln | CAA(Q) | 78 | **1.51** |
|  | CUG(L) | 16 | 0.18 |  | CAG(Q) | 25 | 0.49 |
| Ile | AUU(I) | 300 | **1.78** | Asn | AAU(N) | 160 | **1.80** |
|  | AUC(I) | 38 | 0.22 |  | AAC(N) | 18 | 0.20 |
| Met | AUA(M) | 188 | **1.51** | Lys | AAA(K) | 107 | **1.54** |
|  | AUG(M) | 61 | 0.49 |  | AAG(K) | 32 | 0.46 |
| Val | GUU(V) | 71 | **2.17** | Asp | GAU(D) | 89 | **1.85** |
|  | GUC(V) | 14 | 0.43 |  | GAC(D) | 7 | 0.15 |
|  | GUA(V) | 31 | 0.95 | Glu | GAA(E) | 76 | **1.49** |
|  | GUG(V) | 15 | 0.46 |  | GAG(E) | 26 | 0.51 |
| Ser(UCN) | UCU(S) | 60 | **2.08** | Cys | UGU(C) | 30 | **1.71** |
|  | UCC(S) | 20 | 0.69 |  | UGC(C) | 5 | 0.29 |
|  | UCA(S) | 56 | 1.94 | Trp | UGA(W) | 59 | **1.57** |
|  | UCG(S) | 8 | 0.28 |  | UGG(W) | 16 | 0.43 |
| Pro | CCU(P) | 26 | **1.93** | Arg | CGU(R) | 9 | **2.25** |
|  | CCC(P) | 12 | 0.89 |  | CGC(R) | 0 | 0.00 |
|  | CCA(P) | 14 | 1.04 |  | CGA(R) | 6 | 1.50 |
|  | CCG(P) | 2 | 0.15 |  | CGG(R) | 1 | 0.25 |
| Thr | ACU(T) | 51 | **2.22** | Ser(AGN) | AGU(S) | 34 | **1.18** |
|  | ACC(T) | 10 | 0.43 |  | AGC(S) | 4 | 0.14 |
|  | ACA(T) | 24 | 1.04 |  | AGA(S) | 32 | 1.11 |
|  | ACG(T) | 7 | 0.30 |  | AGG(S) | 17 | 0.59 |
| Ala | GCU(A) | 17 | **2.43** | Gly | GGU(G) | 44 | **2.00** |
|  | GCC(A) | 1 | 0.14 |  | GGC(G) | 0 | 0.00 |
|  | GCA(A) | 9 | 1.29 |  | GGA(G) | 16 | 0.73 |
|  | GCG(A) | 1 | 0.14 |  | GGG(G) | 28 | 1.27 |


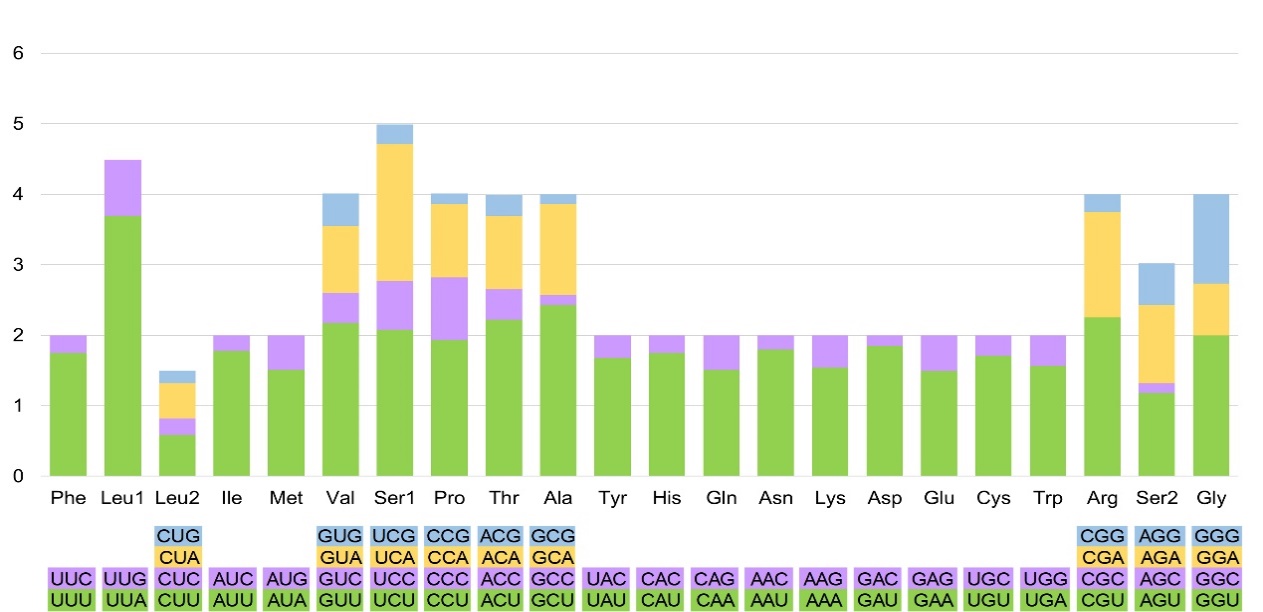
Fig S1. The relative synonymous codon usage (RSCU) of the mitochondrial genome of *Binodoxys acalephae*.


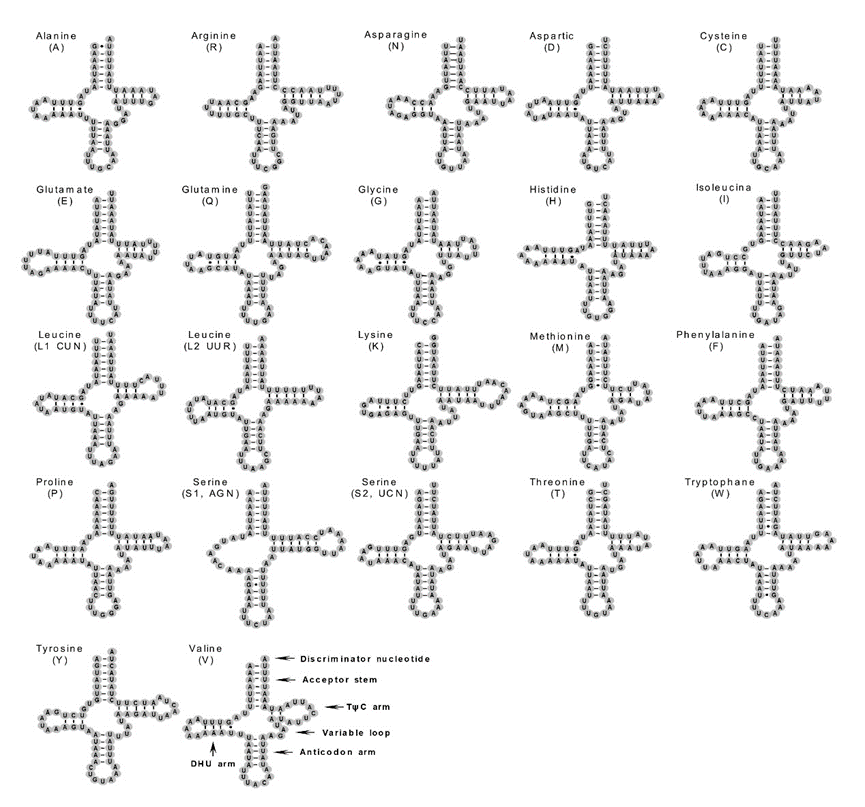
Fig S2. Predicted secondary structures of the 22 tRNA genes of *Binodoxys acalephae*. Short lines indicate Watson–Crick base-pairing and dots indicate noncanonical G-U pairs.
